# Unveiling the Knowledge Gap Regarding Standard Antibiotic Prophylaxis for Infective Endocarditis Among Physicians, Dentists and Cardiologists in Pakistan: A Cross-Sectional Study

**DOI:** 10.7759/cureus.52938

**Published:** 2024-01-25

**Authors:** Mamoon Qadir, Waqar Mustafa, Awais Ahmed Nizami, Hamid Iqbal, Maria Shahzad, Anwar Ali, Amna Akbar

**Affiliations:** 1 Cardiology, Federal Government Polyclinic, Islamabad, PAK; 2 Cardiology, Combined Military Hospital, Muzaffarabad, PAK; 3 Cardiology, Shahida Islam Medical Complex, Lodhran, PAK; 4 Cardiology, Kulsum International Hospital, Islamabad, PAK; 5 Emergency and Accident, District Headquarter Jhelum, Muzaffarabad, PAK

**Keywords:** current guidelines, prophylaxis, antibiotics, mitral and tricuspid valve, prosthetic valve, infective endocarditis

## Abstract

Introduction: Infective endocarditis is a microbial infection of the endocardial surface of the native and prosthetic valves. This study aimed to evaluate knowledge regarding the current guidelines for antibiotic prophylaxis for infective endocarditis.

Methods: This cross-sectional study was conducted among physicians, cardiologists, and dentists in Rawalpindi and Islamabad. The questionnaire was distributed as Google Forms among the required population, and responses were collected on a Google Response Sheet.

Results: The participants viewed rheumatic heart disease (83.7%) and heart transplant (96.7%) as the most vulnerable conditions that warrant the need for antibiotic prophylaxis. The other questions yielded average responses.

Conclusion: The findings of this study emphasize the importance of adhering to standard guidelines and highlight the need for knowledge of the current guidelines.

## Introduction

Infective endocarditis (IE) is a microbial infection of the endocardial surface, a natural or artificial heart valve, or an implanted cardiac device transmitted through blood [1]. Although novel diagnostic and therapeutic approaches have been developed, the incidence of IE is 5 to 7.9 per 100,000 patients, and the 1-year mortality rate has not changed and is 30% [2], which is worse than that for many malignancies. Clinical management is hampered by logistical challenges, a lack of randomized trials, and persistent disagreements over tropics, such as the use of antibiotic prophylaxis [3]. The average patient age doubled, and the percentage of patients with indwelling cardiac devices increased. Streptococci (18.7%) are no longer the most frequent cause of IE; staphylococci (26.6%), frequently associated with medical contacts and invasive procedures, have taken over. Healthcare-associated IE accounts for 25-30% of the IE burden [2].

IE is a fatal disease. Disparities in age, sex, and recent temporal patterns were observed in the incidence and mortality of IE. For example, the prevalence of IE is increasing among young adults in Finland. Compared with women, males, particularly middle-aged men, are more susceptible to IE. The 30-day mortality rate increased with age and remained constant at 11% in Finland, which was the same for both sexes [4].

In a cohort of Indian individuals, the independent predictors of mortality in IE cases included congestive heart failure, a rise in peak leukocyte count, and stroke. In the study population, hemolytic streptococci were the most common microorganisms, and the most prevalent ailment was rheumatic heart disease [5]. Knowledge of the conditions for which antibiotic prophylaxis is recommended, such as rheumatic heart disease, requires further research and education for clarity in recommendations.

## Materials and methods

The study followed a cross-sectional design and was conducted in all tehsil and district hospitals in Rawalpindi and Islamabad and private sector hospitals between December 2022 and September 2023. This study aimed to evaluate the knowledge of physicians, cardiologists, and dentists about the current guidelines regarding the treatment of endocarditis. The sample size was calculated using Cochran's formula: n=z2.p(1-p)/e2. The value of n was 385, which means that we needed to obtain a sample of 385 to reach any acceptable results, where p = 0.5, e = 0.05, and z = 1.96, for a confidence interval of 95%. We approached all the practicing physicians, cardiologists, and dentists in the twin cities, and through the consecutive sampling method, we obtained a sample size of 395. All participants were from Pakistan, were above 18 years old, and could read English. The questionnaire was developed based on the latest guidelines for endocarditis prevention and treatment [6]. The questionnaire was simple and in understandable language. The first part of the questionnaire contained twelve questions that were mainly related to the incidence or prevalence of conditions associated with endocarditis, for example, the incidence of infected cardiac devices and mortality. These incidents are mentioned in the guidelines we followed for the study. The second part of the questionnaire contained the names of lesions commonly associated with endocarditis and the conditions requiring antibiotic prophylaxis.

The data was collected using Google Forms links to every physician, cardiologist, and dentist working in the two towns mentioned above. All social media sources (WhatsApp, Telegram, and Messenger) were used to ensure the maximum number of health professionals was reached. The data were transferred to a Microsoft Excel sheet, and basic analysis was performed. Missing values were replaced with adjacent column values in case the performa had one or two missing values. Incomplete performas were not included in the data set and were deleted at the initial step. The frequencies and percentages were determined after transferring the data to SPSS 25.0.

## Results

In this cross-sectional study, we surveyed 395 participants to investigate various aspects of infective endocarditis (IE) and cardiac implantable electronic devices (CIED). Most respondents (78.5%) indicated that echocardiography was the first-line imaging modality for suspected IE cases. The respondents identified echocardiography as the primary method to confirm native valve endocarditis, with 86.2% (n = 340) agreeing. Among participants, 67.3% (n = 266) agreed that elective interventions like respiratory, dental, gastrointestinal, and genitourinary should be avoided in the presence of fever or other infection symptoms after prosthetic heart valve placement. According to the 2023 study on PVE-IE, the exact prevalence of IE was 37.8% among cardiologists and physicians. The correct PVE-IE-associated mortality rate, according to the 2023 study, was 23.4% among the study participants. 48.9% believed that surgical intervention for TAVI-IE could be performed within 4-6 weeks after diagnosis. 45.5% (n = 180) knew the prevalence of transcatheter pulmonary valve IE (TPV-IE), and 17.2% of participants (n = 68) knew the mortality rate associated with TPV-IE.

The standard protocol for prophylaxis in the presence of cardiac implantable electronic devices related to IE (CIED-IE) involved the administration of antibiotics, which was reported correctly by 62.5% (n = 247) of the participants. 55.1% (n = 218) of respondents indicated that the protocol for dealing with an infected device in the case of CIED-IE involves device removal and subsequent antibiotic therapy. Most participants (79.3%, n = 313) recommended a course of antibiotic therapy for CIED-IE lasting 4-6 weeks (Table 1). For 77.2% of people who had a prosthetic heart valve or surgery to fix the valve, transcatheter mitral and tricuspid valve repair (61.0%), previous infective endocarditis (74.4%), or a heart transplant (96.7%), antibiotics were also needed. Of the respondents, 65.3% believed that high-risk patients undergoing invasive diagnostic and therapeutic procedures for the respiratory, musculoskeletal, skin, genitourinary, and gastrointestinal systems require antibiotic prophylaxis.

**Table 1:** General Knowledge about Infective Endocarditis Note: The correct answer is given in supplementary table

Response	Correct	Percentage
I consent to participate in this study	395	100%
What is the first line imaging modality for suspected IE cases	85	21.5%
Native valve endocarditis can be confirmed with	340	86.2%
Respiratory, dental, gastrointestinal, and genitourinary elective interventions should be avoided in presence of fever or other infection symptoms after placement of prosthetic heart valve?	266	67.3%
What is prevalence of prosthetic valve endocarditis (PVE-IE)	149	37.8%
Prosthetic valve endocarditis PVE-IE associated mortality rate	92	23.4%
Transcatheter prosthetic aortic valve endocarditis (TAVI-IE) incidence	193	48.9%
Surgical intervention for Transcatheter prosthetic aortic valve endocarditis (TAVI-IE) can be done within	193	48.9%
Transcatheter pulmonary valve endocarditis (TPV-IE) incidence	180	45.5%
Transcatheter pulmonary valve endocarditis (TPV-IE) associated mortality	68	17.2%
What is standard protocol of prophylaxis for procedures in presence of cardiac implantable electronic devices related IE (CIED-IE)?	247	62.5%
What is the protocol for an infected device in case of CIED-IE?	218	55.1%
What is recommended time for antibiotic regimen for cardiac implantable electronic devices related IE (CIED)?	313	79.3%
Note: The correct answer is given in supplementary table		

**Figure 1:**
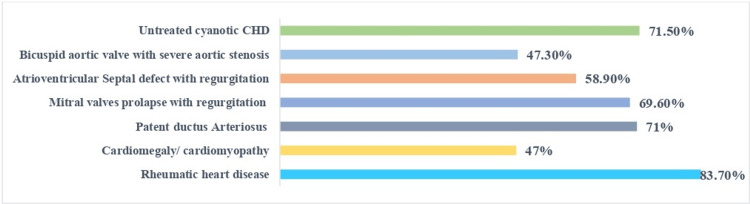
The percentage of healthcare providers who think antibiotic prophylaxis for cardiac lesions is essential Each cardiac lesion got a different response, from 47% for cardiomyopathy to 83.7% for rheumatic heart disease..

The respondents identified the following conditions for which antibiotic prophylaxis is recommended (practice): 83.7% selected rheumatic heart disease (n=305), 69.6% chose mitral valve prolapse with regurgitation (n=275), and 58.9% thought that atrioventricular septal defect with regurgitation (n=233) needed antibiotic prophylaxis. Of the participants, 47.3% thought that bicuspid aortic valve with severe aortic stenosis (n=187) and untreated cyanotic CHD (71.5%, n=282) also required antibiotics. The participants agreed that antibiotic prophylaxis before oral dental procedures was recommended (Figure 1).

Prosthetic cardiac valve and surgical repair of the valve (77.2%), transcatheter mitral and tricuspid valve repair (61.0%), previous infective endocarditis (74.4%), and heart transplant (96.7%) also required antibiotics. Of the respondents, 65.3% believed that high-risk patients undergoing invasive diagnostic and therapeutic procedures for the respiratory, musculoskeletal, skin, genitourinary, and gastrointestinal systems require antibiotic prophylaxis (Table 2).

**Table 2:** The response for need of antibiotics for different cardiac lesions. The healthcare providers responded about the need for antibiotics in particular conditions or lesions

Response	Correct	Percentage
The lesions for which antibiotic prophylaxis is recommended		
Rheumatic heart disease	331	83.7%
Cardiomegaly/ cardiomyopathy	185	47%
Patent ductus Arteriosus	280	71%
Mitral valves prolapse with regurgitation	275	69.6%
Atrioventricular Septal defect with regurgitation	233	58.9%
Bicuspid aortic valve with severe aortic stenosis	187	47.3%
Untreated cyanotic CHD	282	71.5%
Oro dental procedures like extraction and other procedures requiring incision in presence of		
Prosthetic cardiac valve and surgical repair of the valve	305	77.2%
Transcatheter mitral and tricuspid valve repair	241	61.0%
Previous infective endocarditis	294	74.4%
Heart transplant	382	96.7%
High risk patients undergoing invasive diagnostic and therapeutic procedures of respiratory, musculoskeletal, skin, genitourinary and gastrointestinal systems	258	65.3%

## Discussion

The results of this cross-sectional study shed light on several crucial aspects related to infective endocarditis (IE) and cardiac implantable electronic device-related infections (CIED-IE). The high preference for echocardiography as the first-line imaging modality for suspected IE cases aligns with the established clinical guidelines. Echocardiography offers real-time visualization of cardiac structures, aiding the timely diagnosis of endocarditis. The reliance on blood culture and echocardiography to confirm native valve endocarditis reflects the importance of a multifaceted diagnostic approach. Accurate and early diagnosis is critical for prompt initiation of appropriate treatment. The consensus on avoiding elective interventions in the presence of fever or infection symptoms after prosthetic heart valve placement emphasizes the need for caution. Infection prevention is paramount in this vulnerable population, as elective procedures can introduce pathogens and exacerbate the risk of endocarditis.

The usual way to prevent CIED-IE, which involves giving antibiotics, is in line with established guidelines (European Society of Cardiology (ESC) Guidelines for the Management of Endocarditis: Developed by the Task Force on the Management of Endocarditis). Effective managing CIED-related infections, including device removal and antibiotic therapy, is crucial to preventing complications.

The recommended 4-6 weeks duration for antibiotic therapy in patients with CIED-IE reflects the complexity of managing device-related infections. Balancing the need for adequate treatment and minimizing the risk of complications is essential. Knowledge of the conditions for which antibiotic prophylaxis is recommended, such as rheumatic heart disease, requires further research and education for clarity in recommendations. The consensus on antibiotic prophylaxis before oro-dental procedures, particularly in high-risk populations, aligns with the established guidelines to prevent infective endocarditis. Educating patients and healthcare providers is essential to ensuring adherence to these recommendations. Recognition of the need for antibiotic prophylaxis in high-risk patients undergoing various invasive procedures highlights the importance of risk assessment and tailored prevention strategies. Surgery was performed on 3143 patients (73.1%) between 1994 and 2016 for native valve endocarditis and 1157 patients (26.95%) for prosthetic valve endocarditis. Compared to native valve endocarditis, patients who underwent surgery for prosthetic valve endocarditis had considerably lower long-term survival rates, according to the Kaplan-Meier analysis (p = 0.001). After multivariable adjustment, there was no discernible difference between patients who underwent cardiac surgery with prosthetic valve endocarditis and native valve endocarditis in terms of long-term survival [7]. Of 395 respondents in our study, 48.9% recommended surgery for endocarditis.

A systematic review found that the understanding of guidelines ranged from 1.9% to 100%, depending on the specifics being examined. The expertise of the dental students and licensed dentists did not differ significantly. Guidelines were followed, on average, between 40% and 81% of the time. The factors most strongly correlated with respondents' understanding of IE criteria for infective endocarditis were age and postgraduate training. Research on dentists' and dental students' adherence to recommendations for preventing infective endocarditis has not been published sufficiently [8]. Patients with artificial valves (78.4%) had a history of IE (77%), and others were prescribed antibiotics. The most common intervention recommended by responders was tooth extraction (70.7%). The most popular medication was amoxicillin (63.5%), and clindamycin was the antibiotic of choice for allergic patients (55.4). Although the choice of antibiotics was made in accordance with the American Heart Association (AHA) guidelines from 2008, most dentists (58.82 and 55.4%) were unaware of the proper dosage and timing of azithromycin and clindamycin administration to patients who had penicillin allergies [9]. Dentists in the Aseer Region have little awareness of the use of preventive medications in managing and preventing IE. The most recent AHA recommendations should be incorporated into dental school curricula [10]. Our study reported knowledge about guidelines correctly by 23.4% to 96.7% of participants.

Although not all clinical questions were correctly answered, most respondents (76%) answered correctly. It is important to stress that dentistry students may never receive training on antimicrobial stewardship (AMS) in the future. It would be best if this problem is addressed in dental schools. Potential nationalization of dental AMS education delivery across Australia is one method to combat this [11]. Healthcare workers need to be aware of the prophylaxis needs cases. Antibiotic stewardship should be at the forefront of patient care because the overuse of antibiotics contributes to the emergence of drug resistance. Training programs must include this knowledge [12]. The same suggestion was given by another researcher [13]; it is difficult for practitioners to recognize individuals who are at high risk for cardiac diseases and dental procedures that are known to induce bacteremia [14]. The participants in our study also had average knowledge of the high-risk population.

The American Dental Association has defined standards dentists should adhere to when prescribing antibiotic prophylaxis. It is important to educate them on the importance of precise oral hygiene and to schedule routine dental examinations to maintain their dental health [15]. Most of the respondents (68.8%), family doctors (2.6%), and infectious disease experts (1.3%) were consulted when deciding on IE prophylaxis and 3.9% of them implemented prescriptions solely based on their professional experience [16]. However, this study also contradicts this [17]. Among the participants, 82.1% expressed a medium level of knowledge of prophylactic treatment [18]. An important aspect of IE prevention is the knowledge of patients seeking treatment [19]. Our study did not involve the participants from the general population. The highest percentage of participants voted for heart transplant and rheumatic fever as conditions that warranted antibiotic prophylaxis.

Patients thought to be at high risk of infective endocarditis (patients with past infective endocarditis heart valve prosthesis and certain congenital heart problems) are advised to receive antibiotic prophylaxis [20]. Over a century ago, dental procedures and Infective Endocarditis (IE) were presumed to be related. Soon after, medical professionals began attempting to reduce the risk of IE by using antibiotics. Since then, there have been discussions about the efficiency of antibiotic prophylaxis (AP) and the possibility that invasive dental procedures contribute to the emergence of IE. The lack of prospective randomized clinical trial data has been a major contributing factor to this dispute. From this unsatisfactory position, guidelines committees representing many groups and nations have faith in reaching an ideal position on whether AP usage is required for invasive dental operations (or other procedures) and for whom. The following are the results of a study involving a substantial number of patients with US. The study showed a significant temporal relationship between invasive dental treatments and the onset of IE in high-IE-risk patients by utilizing a cohort and case-crossover methodology. This study also revealed that AP use was associated with a lower risk of IE. Additional findings from a different study conducted by Thornhill et al. using national hospital admission data from England were also released this year, demonstrating the substantial correlation between the development of IE and the number of dental and non-dental treatments. Two more investigations have raised similar concerns regarding non-invasive dental procedures and IE risk. Collectively, the findings of this research encourage reassessment of the National Institute for Health and Care Excellence (NICE) and other organizations [21].

The 2007 American Heart Association (AHA) recommendations restricted the use of antibiotic prophylaxis (AP) for infective endocarditis (IE) to fewer patients with congestive cardiac conditions. The American Academy of Pediatrics Section on Cardiology and Cardiac Surgery (AAP SOCCS) was surveyed regarding suggestions for AP for various PCCs and procedures. We present the data of 173 responders who adhered to the 2007 AHA recommendations. The AP rates for high-risk PCCs that clearly met the AHA criteria ranged between 70.8% and 89.8%. In contrast, the prescription rates ranged from 1% to 29.5% for PCCs that did not meet the AHA standards. According to these rules, the PCC's AP indication was imprecise, and its AP rates ranged from 9.9 to 39.8%. Similar variations in AP have been observed in several procedures in high-risk PCC settings. The variation in antibiotic prophylaxis (AP) prescribing policies among pediatric cardiologists, particularly in the context of both underlying pediatric congenital cardiac (PCC) problems and non-cardiac procedures, is an important issue that requires careful study [22].

Infectious endocarditis, a rare but lethal condition, has an annual incidence of 5-10 per 100,000 people. Therefore, antibiotic prophylaxis is crucial for preventing this condition. However, the justification for using antibiotic prophylaxis to help prevent IE is also controversial because there is no firm proof of its effectiveness. It is important to discuss whether the antibiotic prophylaxis regimen suggested for preventing infective endocarditis in individuals with periodontitis is sufficient. Elderly people without obvious valve illness are now the group most at risk for infection, not young people with documented rheumatic valvular heart disease. A thorough systematic evaluation is required to evaluate whether the current limitations on using AP are still necessary [23]. Dental implant surgery, periodontal surgery, extraction of teeth from patients with poor oral hygiene, and minor surgery for an impacted tooth with a recent bout of infection were assessed as presenting a moderate-to-high risk for IE. The cardiac diseases that were strongly advised for antibiotic prophylaxis were severe mitral valve stenosis or regurgitation as well as prior IE. Less than half of Malaysian clinical experts concurred with the modifications to the 2008 NICE guidelines, which may have contributed to their insistence that antibiotic prophylaxis is still necessary for high-risk cardiac diseases and invasive dental operations [24]. The prognosis remains poor despite breakthroughs in diagnosis and treatment, and a rise in morbidity and hospital mortality underlines the importance of an early diagnosis of this illness. Given its intricacy, infective endocarditis patients require an immediate reaction, swift diagnosis, and treatment from a multidisciplinary team with a speedy diagnostic methodology. The timing of surgery and the function of antibiotic prophylaxis in management remain unproven. It is important to study the epidemiological shift, diagnostic difficulties, and emerging approaches for treating infective endocarditis medically and surgically [25].

The study has certain limitations. It was conducted shortly after the AHA guidelines (2023) were published, and some health professionals have probably not read the latest update. Secondly, the study did not have a homogenous sample, as the respondents belonged to general physicians, cardiologists, and dentists. Most respondents did not mention their department, so we entirely skipped mentioning the professional details. Additionally, the data collection is limited to only two cities because the data curators were residing in these two cities of Pakistan, and they reached people practicing there.

## Conclusions

In conclusion, this study provides valuable insights into the current practices and recommendations related to IE and CIED-IE among healthcare professionals. These findings emphasize the importance of adhering to established guidelines for diagnosing, managing, and preventing these infections. Additionally, the study identified areas where further research and guideline refinement are warranted, particularly in the context of transcatheter valve procedures and antibiotic prophylaxis recommendations. These results can inform clinical practice, ultimately improving patient outcomes in the field of cardiology and infectious diseases.

## Appendices

**Table 3:** Supplementary Table

Questions	Correct Response
What is the first line imaging modality for suspected IE cases	Echocardiography
	Transthoracic Echocardiography
Native valve endocarditis can be confirmed with	Blood culture
	Echocardiography
Respiratory, dental, gastrointestinal, and genitourinary elective interventions should be avoided in presence of fever or other infection symptoms after placement of prosthetic heart valve?	Yes
What is prevalence of prosthetic valve endocarditis (PVE-IE)	20–30%
Prosthetic valve endocarditis PVE-IE associated mortality rate	23–64%
Transcatheter prosthetic aortic valve endocarditis (TAVI-IE) incidence	Up to 1.9/100 patients’ years
Surgical intervention for Transcatheter prosthetic aortic valve endocarditis (TAVI-IE) can be done within	1 – 3 months
Transcatheter pulmonary valve endocarditis (TPV-IE) incidence	1.6 – 4/100 patient years
Transcatheter pulmonary valve endocarditis (TPV-IE) associated mortality	0 – 11%
What is standard protocol of prophylaxis for procedures in presence of cardiac implantable electronic devices related IE (CIED-IE)?	Give systemic antibiotics within one hour of incision
What is the protocol for an infected device in case of CIED-IE?	Remove the device completely
What is recommended time for antibiotic regimen for cardiac implantable electronic devices related IE (CIED)?	4- 6 weeks
